# Supplementary material for: Predictors of the Third (Booster) Dose of COVID-19 Vaccine Intention among the Healthcare Workers in Saudi Arabia: An Online Cross-Sectional Survey
Source: Vaccines (Basel). 2022 Jun 21;10(7):987. doi: 10.3390/vaccines10070987 (PMC9317226; doi:10.3390/vaccines10070987)
Supplement: Supplementary file 1 [file vaccines-10-00987-s001.zip › vaccines-1761752-supplementary.pdf]

Predictors of Intent to Receive the third dose (booster) of COVID-19 Vaccine among among health care workers/مدى تقبل الممارسين الصحيين لتلقي جرعة التطعيم الثالثة المعززة ضد فيروس كورونا

This is an online survey (will take around 3 minutes) to assess the predictors of intent to receive the third dose (booster) of COVID-19 vaccine among health care workers in Saudi Arabia. This survey is anonymous, the identity of the participants shall not be revealed.
Principal investigator:
Department of Internal Medicine
Faculty of Medicine
University of Jeddah

السلام عليكم ورحمة الله, هذه الاستبانة ضمن دراسة عن تقبل الممارسين الصحيين في السعودية لتلقي جرعة التطعيم الثالثة المعززة ضد فيروس كورونا المستجد. المشاركة في هذا الاستطلاع تطوعية تمامًا وتستغرق تقريبا 3 دقائق وجميع ردودك بدون اسم ومجهولة الهوية
الباحث الرئيسي:
قسم الطب الباطني
كلية الطب
جامعة جدة

* Required

1. I'm a health care worker and I agree to participate/ انا ممارس صحي وأوافق على المشاركة *

Mark only one oval.

- ( ) Yes/نعم
- ( ) No/لا

Untitled Section

2. Have you been vaccinated for COVID-19/ هل تلقيت لقاح فيروس كورونا المستجد *

Mark only one oval.

- Yes, completed 3 doses/نعم وتلقيت الجرعة الثالثة
- Yes, completed 2 doses/نعم وتلقيت التطعيمتين
- Yes, received one dose only/نعم وتلقيت تطعيمة واحدة فقط
- No/لا

3. What type of vaccine have you received? ماهو نوع اللقاح الذي تلقيته؟ *

Mark only one oval.

- Pfizer/BioNTech/ فايزر بيونتيك
- Oxford/AstraZeneca/ استرازينيكا اكسفورد
- Moderna/موديرنا
- MIX (2 different vaccines)/ نوعين مختلفين
- I don't know or not sure/لا أعلم او غير متأكد

Section A: GENERAL INFORMATION/ معلومات عامة

4. 1. Age in years / العمر بالسنوات *

____

5. 2. Gender /الجنس *

Mark only one oval.

- a. Male/ذكر
- b. Female/ أنثي

6. 3. Nationality / الجنسية *

Mark only one oval.

- a. Saudi/سعودي
- b. Non Saudi/غير سعودي

7. 4. Marital Status / /الحالة الاجتماعية *

Mark only one oval.

- a. Married/متزوج
- Widow/divorced/seperated/منفصل/مطلق/ارمل
- /Single/never married/ لم يتزوج قط / أعزب

8. 5.Religion/الديانة *

Mark only one oval.

- 1. Muslim/ مسلم
- 2. Christian/مسيحي
- 3. Other/اخرى

9. 6. Highest education /التعليم *

Mark only one oval.

- a. High school and below/ تعليم ثانوي أو أقل
- b. Bachelor or diploma/ بكالوريوس او دبلوم
- c. Master or PhD/ ماجستير أو دكتوراه

10. 7. Occupation / المهنة *

Mark only one oval.

- Dentist/طبيب اسنان
- Nurse/ممرض
- Physician/طبيب
- Pharmacist /صيدلي
- Technician/فني
- Other: ____________________

11. 8. I'm working in/انا أعمل في *

Mark only one oval.

- Hospital/مستشفى
- Outpatient clinic, lab or radiology/مركز عيادات, مختبر او أشعة خارجية
- outpatient procedure unit (dialysis Center, Endoscopy unit or day surgery unit)/وحدات خارجية (مركز غسيل الكلى ، وحدة المناظير أو وحدة جراحة اليوم الواحد)
- long term facility/Home care/الرعاية المنزلية / مرفق رعاية طويلة الأمد /
- Other: ____________________

12. 9. Average monthly income (SR) (متوسط الدخل الشهري (ريال سعودي *

Mark only one oval.

- a. Less than 5,000 أقل من
- b. 5001-10,000
- c. 10001-15000
- d. More than15,000 اكثر من

13. 10. Current location (province)/ الموقع الحالي(المنطقة) *

Mark only one oval.

- Central Region/المنطقة الوسطى
- Eastern region/المنطقة الشرقية
- Northern region/المنطقة الشمالية
- Southern region/المنطقة الجنوبية
- Western region/المنطقة الغربية

14. 11. Are you a smoker?/هل انت مدخن؟ *

Mark only one oval.

- Yes/نعم
- No/لا
- ex-smoker/مدخن سابق
- Passive smoker/مدخن سلبي(استنشاق دخان تبغ الآخرين)

15. 12. Physical activity/ النشاط البدني *

Mark only one oval.

- Sedentary lifestyle(none)/لا أقوم بأي نشاط رياضي بصفة دورية
- < 150 min. / week/ نشاط رياضي(اقل من 150 دقيقة اسبوعيا)
- >150 min./week/نشاط رياضي(اكثر من 150 دقيقة اسبوعيا)

16. 13. In your daily work, are you dealing directly with patients who are suspected or confirmed diagnosis of COVID-19? هل تتعامل مباشرة مع مرضى يشتبه في إصابتهم بـكوفيد -19 أو تأكد تشخيصهم؟ *

Mark only one oval.

- a. Yes/ نعم
- b. No/لا

Previous COVID-19 illness/(الإصابة بكوفيد -19 (فيروس كورونا المستجد

17. 14. Have you ever been ill with COVID-19 in the past? هل سبق ان أصبت بفيروس كورونا المستجد؟ *

Mark only one oval.

- a. Yes/ نعم
- b. No/لا

18. 15. Has any of your close family members been ill with COVID-19? هل أصيب أحد أفراد أسرتك المقربين بفيروس كورونا المستجد؟ *

Mark only one oval.

- a. Yes/ نعم
- b. No/لا

General Health/الصحة العامة

19. 16. Do you have an existing chronic disease that requires you to take chronic medications?هل تعاني من مرض مزمن يتطلب منك تناول أدوية بصفة دورية؟ *

Mark only one oval.

- a. Yes/ نعم
- b. No/لا

20. 17. How do you rate your overall health?كيف تقيم صحتك العامة؟ *

Mark only one oval.

- Very good/جيدة جدا
- Good/جيدة
- Fair/ مقبولة
- Poor/سيئة
- Very poor/سيئة جدا

Section B: ACCEPTABILITY OF COVID-19 VACCINATION WHEN AVAILABLE. تقبل التطعيم ضد فيروس كورونا المستجد

21. 18. If Saudi MOH recommends health care worker to take the 3rd dose (booster) of COVID-19 vaccine, Are you planning to take it ?لو أوصت وزارة الصحة الممارسين الصحيين بأخذ جرعة التطعيم الثالثة المعززة ضد فيروس كورونا, هل تخطط لأخذها ؟ *

Mark only one oval.

- a. Definitely no/قطعا لا
- b. Probably no/على الأرجح لا
- c. Possibly yes/ربما نعم
- d. Definitely yes/قطعا نعم

22. 19. Rate your confidence in using locally manufactured COVID-19 vaccine: في حال تم تصنيع لقاح محلي لفيروس كورونا المستجد، قيم ثقتك في استخدام هذا اللقاح؟ *

Mark only one oval.

- a. Completely confident/ واثق تماما
- b. Confident/ واثق
- c. Not confident/ غير واثق
- d. Completely not confident/غير واثق تماما

23. 20. Rate your confidence in using foreign-manufactured (imported) COVID-19 vaccine: قيم ثقتك في استخدام لقاح أجنبي الصنع (مستورد) لفيروس كورونا المستجد *

Mark only one oval.

- a. Completely confident/ واثق تماما
- b. Confident/ واثق
- c. Not confident/ غير واثق
- d. Completely not confident/غير واثق تماما

24. 21. Please indicate your preferences in local/imported COVID-19 vaccine: يرجى تحديد اللقاح المفضل لديك *

Mark only one oval.

- a. I prefer local manufactured COVID-19 vaccine/أنا أُفَضِّلْ لقاح محلي الصنع
- b. I prefer imported/foreign manufactured COVID-19 vaccine أنا أُفَضِّلْ لقاح مستورد (أجنبي الصنع)
- c. I don’t have preference as long as there is vaccine available/ليس لدي أفضلية طالما كان هناك لقاح متوفر

Section D: HEALTH BELIEF MODEL نموذج الاعتقاد الصحي

Perceived susceptibility of contacting COVID-19 احتمالية الإصابة بفيروس كورونا المستجد المتوقعة

25. 22. My chance of getting COVID-19 in the next few months is high/امكانية إصابتي بفيروس كورونا المستجد في الأشهر القليلة المقبلة عالية *

Mark only one oval.

- a. Strongly agree/ موافق بشدة
- b. Agree/موافق
- c. Disagree/معارض
- d. Strongly disagree/معارض بشدة

26. 23. I am worried about the likelihood of getting COVID-19.أنا قلق من احتمال الإصابة بفيروس كورونا المستجد *

Mark only one oval.

- a. Strongly agree/ موافق بشدة
- b. Agree/موافق
- c. Disagree/معارض
- d. Strongly disagree/معارض بشدة

27. 24. Getting COVID-19 is currently a possibility for me. احتمال الإصابة بفيروس كورونا المستجد وارد بالنسبة لي *

Mark only one oval.

- a. Strongly agree/ موافق بشدة
- b. Agree/موافق
- c. Disagree/معارض
- d. Strongly disagree/معارض بشدة

Perceived severity of COVID-19. توقعات خطورة الإصابة بفيروس كورونا المستجد

28. 25. Complications of COVID-19 are serious. تعد مضاعفات فيروس كورونا المستجد خطيرة *

Mark only one oval.

- a. Strongly agree/ موافق بشدة
- b. Agree/موافق
- c. Disagree/معارض
- d. Strongly disagree/معارض بشدة

29. 26. I will be very sick if I get COVID-19. إذا أصبت لا سمح الله بفيروس كورونا المستجد، سأكون مريضًا جدًا (أعراض قوية) *

Mark only one oval.

- a. Strongly agree/ موافق بشدة
- b. Agree/موافق
- c. Disagree/معارض
- d. Strongly disagree/معارض بشدة

30. 27. I am afraid of getting COVID-19. أخشى من الإصابة بفيروس كورونا المستجد *

Mark only one oval.

- a. Strongly agree/ موافق بشدة
- b. Agree/موافق
- c. Disagree/معارض
- d. Strongly disagree/معارض بشدة

Perceived benefits of third dose (booster) of COVID-19 vaccine. الفوائد المتوقعة من جرعة التطعيم الثالثة المعززة ضد فيروس كورونا

31. 28.Third dose (booster) of COVID-19 vaccine is a good idea because I will not have to worry about catching COVID-19. جرعة التطعيم الثالثة المعززة فكرة جيدة لأنني لن أقلق بشأن الإصابة بفيروس كورونا المستجد *

Mark only one oval.

- a. Strongly agree/ موافق بشدة
- b. Agree/موافق
- c. Disagree/معارض
- d. Strongly disagree/معارض بشدة

32. 29. Receiving the third dose (booster) of COVID-19 vaccine decreases my chance of getting COVID-19 or its complications. اخذ جرعة التطعيم الثالثة المعززة ضد فيروس كورونا يقلل من احتمالية إصابتي بفيروس كورونا المستجد اومضاعفاته *

Mark only one oval.

- a. Strongly agree/ موافق بشدة
- b. Agree/موافق
- c. Disagree/معارض
- d. Strongly disagree/معارض بشدة

Perceived barriers of third dose (booster) of COVID-19 vaccine. العوائق المتوقعة من تلقي جرعة التطعيم الثالثة المعززة ضد فيروس كورونا

33. 30. The possible side-effects of the third dose (booster) of vaccine would interfere with my usual activities. الآثار الجانبية المحتملة بعد تلقي جرعة التطعيم الثالثة المعززة ستؤثر على حياتي اليومية *

Mark only one oval.

- a. Strongly agree/ موافق بشدة
- b. Agree/موافق
- c. Disagree/معارض
- d. Strongly disagree/معارض بشدة

34. 31. I am concerned about the efficacy of the third dose (booster) of the vaccine. انا قلق من عدم فعالية جرعة التطعيم الثالثة المعززة ضد فيروس كورونا *

Mark only one oval.

- a. Strongly agree/ موافق بشدة
- b. Agree/موافق
- c. Disagree/معارض
- d. Strongly disagree/معارض بشدة

35. 32. I am concerned about the safety of the third dose (booster) of the vaccine. انا قلق بشأن سلامة جرعة التطعيم الثالثة المعززة ضد فيروس كورونا (التطعيم غير آمن) *

Mark only one oval.

- a. Strongly agree/ موافق بشدة
- b. Agree/موافق
- c. Disagree/معارض
- d. Strongly disagree/معارض بشدة

36. 33. I am concerned about the faulty/fake COVID-19 vaccine. انا قلق بشأن تلقي لقاح مزيف او خاطئ (فاسد او غير فعال) ضد فيروس كورونا *

Mark only one oval.

- a. Strongly agree/ موافق بشدة
- b. Agree/موافق
- c. Disagree/معارض
- d. Strongly disagree/معارض بشدة

Cues to action. الدوافع لتلقي اللقاح

37. 34. I will only take the third dose (booster) of COVID-19 vaccine if I was given adequate information about it. سوف أتلقى جرعة التطعيم الثالثة المعززة إذا أعطيت معلومات كافية عنها *

Mark only one oval.

- a. Strongly agree/ موافق بشدة
- b. Agree/موافق
- c. Disagree/معارض
- d. Strongly disagree/معارض بشدة

38. 35. I will only take the third dose (booster) vaccine if it is taken by many health care workers in Saudi Arabia/. سوف أتلقى جرعة التطعيم الثالثة المعززة إذا تم أخذ الجرعة الثالثة من قبل الكثير من الممارسين الصحين في السعودية *

Mark only one oval.

- a. Strongly agree/ موافق بشدة
- b. Agree/موافق
- c. Disagree/معارض
- d. Strongly disagree/معارض بشدة

This content is neither created nor endorsed by Google.

Google Forms
